# Frequency of Aerobic Exercise and Association with Pain and Function in Older Adults with Knee Osteoarthritis: A Cross-Sectional Study

**DOI:** 10.3390/life16030405

**Published:** 2026-03-03

**Authors:** Narucha Komolsuradej, Punyaphat Boonlertwanich, Nakrop Klaylian, Kanyawee Hiranchunha, Danunai Malang, Phruksawan Intharak, Pimmada Sangkaew, Suppakorn Bandrapiwat, Siwaluk Srikrajang

**Affiliations:** 1 Department of Family and Preventive Medicine, Faculty of Medicine, Prince of Songkla University, Kanchanavanich Road 15, Songkhla 90110, Thailand; narucha.ko@psu.ac.th; 2 School of Medicine and Health Science, Faculty of Medicine, Prince of Songkla University, Kanchanavanich Road 15, Songkhla 90110, Thailand; 6310310113@email.psu.ac.th (P.B.); 6310310094@psu.ac.th (N.K.); azye2@hotmail.com (K.H.); 6310310066@psu.ac.th (D.M.); phruksawan@gmail.com (P.I.); 310310123@psu.ac.th (P.S.); 6310310162@psu.ac.th (S.B.); 3 Department of Physical Therapy, Faculty of Medicine, Prince of Songkla University, Kanchanavanich Road 15, Songkhla 90110, Thailand

**Keywords:** pain, knee-strengthening, aerobic exercise, knee osteoarthritis, older adults

## Abstract

Knee osteoarthritis is a leading cause of pain and functional limitation in older adults, and exercise is widely recommended as a core component of conservative management; however, the optimal frequency of aerobic and strengthening exercise remains unclear. This cross-sectional study aimed to examine the association between exercise frequency, pain, and functional outcomes in older adults with knee osteoarthritis. Participants aged ≥60 years with a clinical diagnosis of knee osteoarthritis were recruited from a tertiary university hospital in Thailand. Exercise frequency over the previous four weeks was categorized as none, 1–2 times per week, 3–6 times per week, or every day. Outcomes were assessed using the Thai version of the modified Western Ontario and McMaster Universities Osteoarthritis Index (WOMAC) and the Brief Pain Inventory. Nonparametric analyses and multivariable regression analyses adjusted for age, body mass index, and comorbidities were performed. A total of 140 participants were included, of whom 68.6% reported engaging in aerobic exercise, and 56.4% performed knee-strengthening exercises. Higher frequency of aerobic exercise was significantly associated with lower pain severity, reduced pain interference, and better WOMAC total and subdomain scores (*p* < 0.05). A graded association pattern was observed, with the greatest benefits seen in participants performing aerobic exercise 3–6 times per week. No significant associations were identified between knee strengthening exercise frequency and pain and functional outcomes. These findings suggest that frequent aerobic exercise is associated with reduced pain and improved function in older adults with knee osteoarthritis, supporting its role in primary care and rehabilitation management.

## 1. Introduction

Knee osteoarthritis is a major cause of pain, mobility impairment, and disability in older adults. It is characterized by progressive joint degeneration, synovial inflammation, and cartilage loss, leading to pain, stiffness, and reduced physical function [1]. With the ageing global population, the prevalence of symptomatic knee osteoarthritis continues to increase, imposing significant healthcare burdens and negatively affecting the quality of life of older individuals [2,3]. In 2019, the global prevalence of knee osteoarthritis was estimated at approximately 364.6 million cases. Notably, approximately 73% of individuals with osteoarthritis are ≥55 years of age, with the knee being the most affected joint. Effective management strategies for knee osteoarthritis predominantly emphasize conservative, non-pharmacological approaches, among which exercise therapy, including both aerobic and knee-strengthening exercises, is widely suggested as a cornerstone of treatment [1].

Aerobic exercise has been consistently shown to alleviate pain, enhance joint mobility, and improve overall functional capacity in individuals with knee osteoarthritis [4]. Physiologically, aerobic activity promotes beneficial effects, including increased endorphin release, improved circulation, and enhanced joint nutrition, collectively contributing to pain relief and improved mobility [5]. Regular aerobic exercise may positively affect psychological factors such as mood and pain coping strategies, thus reducing pain interference in daily activities [6]. Similarly, knee-strengthening exercises play a crucial role in managing osteoarthritis by specifically targeting muscle strength deficits around the affected joint. Moreover, strengthening exercises help stabilize the knee, reduce abnormal joint loading, and decrease pain symptoms, thereby facilitating improved physical functioning [7,8]. Despite strong evidence supporting the use of both aerobic and strengthening exercises, the optimal frequency required to achieve clinically meaningful improvements in pain and physical function remains unclear. Some studies have proposed moderate exercise frequencies (e.g., three times per week); however, concerns remain that excessive loading may exacerbate symptoms through mechanical overload [4,5,9].

Given these uncertainties, further research is required to determine how different frequencies of aerobic and knee-strengthening exercises influence key clinical outcomes such as pain severity, pain interference, and functional performance in older adults with knee osteoarthritis. To address this gap, this study aimed to examine the association between exercise frequency, pain, and functional outcomes, with the goal of clarifying the potential graded association. Such evidence may help inform the development of precise evidence-based exercise prescriptions that are both effective and sustainable for the long-term management of knee osteoarthritis.

## 2. Materials and Methods

### 2.1. Study Design and Setting

This study employed a cross-sectional design and was conducted at the orthopedic, rehabilitation, and physical therapy clinics of Songklanagarind Hospital between 25 July and 6 August 2024. A total of 140 participants were recruited based on the following inclusion criteria: (1) individuals aged 60 years and older with a confirmed diagnosis of knee osteoarthritis, as recorded by an orthopedist in the Hospital Information System, (2) ability to communicate effectively in Thai, (3) no prior diagnosis of dementia as verified through medical records or caregiver reports, and (4) no evidence of depression as determined by the 2Q screening questionnaire (administered by the researcher during the screening session). Participants were excluded if they were (1) bedridden, (2) had undergone lower-limb amputation, or (3) presented with active cancer, which limited mobility due to pain. All participants provided written informed consent before study enrolment after being informed of the study.

**Figure 1:**
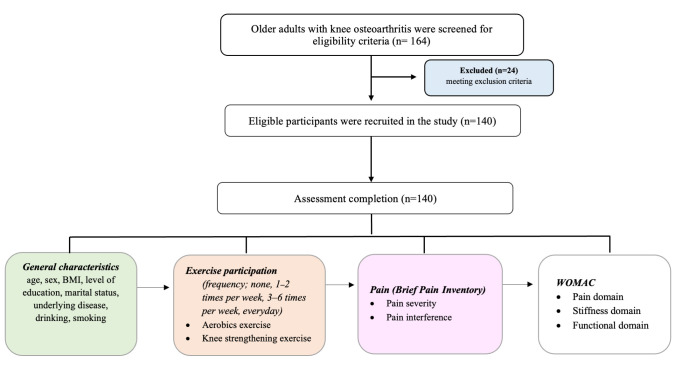
Flowchart of the study (BMI, body mass index; WOMAC, Western Ontario and McMaster Universities Osteoarthritis Index).

The sample size was determined using an a priori power analysis for multiple linear regression (F-test, R^2^ deviation from zero). Assuming a moderate effect size (Cohen’s f^2^ = 0.15), α = 0.05, power = 0.80, and five predictors (exercise participation, age, BMI, dyslipidemia, and cancer), the required minimum sample size was 92 participants. Therefore, the final sample of 140 participants from convenient sampling in this study was considered sufficient, providing an achieved power of approximately 0.95 [10]. The participant flow is presented in Figure 1.

### 2.2. Measurements and Outcomes

The independent variables in this study were the frequency of aerobic exercise and knee-strengthening exercise. Prior to data collection, clear definitions and examples of each exercise type were explained to all participants to ensure consistent understanding. Aerobic exercise was defined as continuous, rhythmic physical activity involving large muscle groups, performed for at least 30 min and at a perceived exertion level described as ‘somewhat hard’ for indication of moderate intensity. Participants were asked: ‘During the past month, how often did you engage in aerobic exercise?’ To facilitate comprehension, ‘somewhat hard’ was explained as corresponding to a Rating of Perceived Exertion between 12 and 14 on the Borg 6–20 scale [11]. Knee-strengthening exercise was defined as any intentional exercise aimed at improving the strength of the muscles surrounding the knee joint to reduce knee pain or enhance function. Participants were asked: ‘During the past month, have you performed knee-strengthening exercises or exercises aimed at reducing knee pain as recommended by a healthcare professional? If yes, please report the frequency per week.’ Standardized examples, including straight leg raises, squats, step-ups, or similar exercises performed with or without resistance, were provided to all participants to support accurate self-reporting [4,8].

The dependent variables were knee pain severity, pain interference, and knee-related functional outcomes, assessed using the Western Ontario and McMaster Universities Osteoarthritis Index (WOMAC) and the Brief Pain Inventory (BPI) questionnaire. Exercise frequency was recorded as the mean exercise frequency over 1 month (4 weeks) and categorized as none, 1–2 times per week, 3–6 times per week, or 7 times per week (every day). Knee pain, stiffness, and knee-related physical function were assessed using the WOMAC Thai version. The instrument consists of 24 items, including 5 items for pain, 2 items for stiffness, and 17 items for physical function. Each item is rated on an 11-point numeric rating scale ranging from 0 (no symptoms) to 10 (extreme symptoms), with higher scores indicating greater symptom severity. Domain scores were calculated as the sum of scores of items within each respective domain. The overall WOMAC scores were calculated as the sum scores of all 24 items, resulting in a total score ranging from 0 to 240. The Thai version of the WOMAC has demonstrated good internal consistency (Cronbach’s alpha ranging 0.85–0.97) and test–retest reliability (correlation coefficient = 0.65–0.71) across all domains [12]. Pain severity and interference were assessed by using the BPI Thai version. The BPI includes two primary domains: pain severity (typically measured through four items rating the worst, least, average, and current pain in the past 24 h) and pain interference (seven items assessing the extent to which pain irritates general activity, mood, walking ability, normal work, relations with others, sleep, and enjoyment of life). The Thai version of the BPI has demonstrated excellent psychometric properties in prior validation studies, with Cronbach’s alpha coefficients ranging from 0.88 to 0.94 across both the severity and interference subscales [13].

Additional health and demographic data were collected through face-to-face interviews, including demographic and health-related variables, such as sex, age, height, weight, marital status, education level, underlying diseases, alcohol consumption, and smoking habits. The 2Q questionnaire was administered to screen for depression as an exclusion criterion. This tool asks whether participants had experienced diminished interest or feelings of sadness or hopelessness in the previous 2 weeks. The 2Q questionnaire is a brief screening tool officially published by the Thai Ministry of Public Health [14].

### 2.3. Statistical Analysis

Data were analyzed using R version 4.4.1. Descriptive statistics were used to summarize participant demographics and health-related variables. Group comparisons were performed using non-parametric tests (Kruskal–Wallis test or Wilcoxon rank-sum test) due to the non-normal distribution of outcome variables.

Multivariable regression models were used to examine the association between exercise participation (independent variable) and WOMAC outcomes (dependent variables), adjusting for age, body mass index, and selected comorbidities. Results are presented as β coefficients with 95% confidence intervals, and statistical significance was set at *p* < 0.05. Covariates were selected a priori based on clinical relevance and prevalence in the study population rather than statistical significance. Age and BMI were included as established determinants of knee osteoarthritis severity, while comorbidities with sufficient prevalence and potential relevance to pain and functional outcomes were retained to ensure model stability.

Exercise participation was treated as a binary categorical variable and entered the regression models; therefore, no assumption of linearity across frequency levels was imposed. Regression assumptions, including linearity, normality of residuals, and multicollinearity, were assessed using standard diagnostic procedures. Variance inflation factors (VIF) were below 5 for all variables, and no substantial violations were observed.

## 3. Results

### 3.1. Participants’ Characteristics

A total of 140 older adults with clinically diagnosed knee OA were included in this study (mean age, 70 years). Most were female (65.7%) and overweight (63.6%) [15]. Aerobic exercise participation was reported by 68.6%, whereas 56.4% performed knee-strengthening exercises at least once per week. Walking and running were the most common aerobic activities (64.3%). Comorbidities included dyslipidemia (51.4%), hypertension (50.7%), and diabetes mellitus (25.7%). Based on pain severity assessed using the Numeric Pain Rating Scale (NPRS), participants were categorized into three pain levels: mild pain (score 1–4; 41.4%), moderate pain (score 5–7; 51.4%), and severe pain (score 8–10; 7.12%) (Table 1).

### 3.2. Exercise Frequency and Pain Outcomes

**Figure 2:**
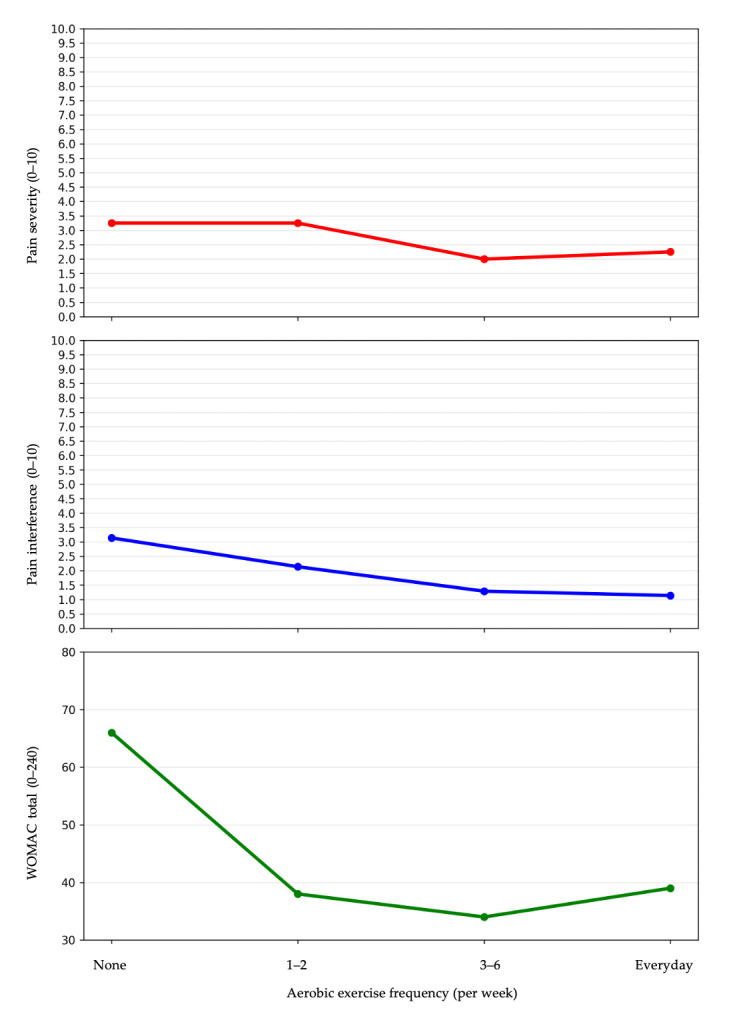
Demonstration of graded association between aerobic exercise frequency and pain severity, pain interference, and total WOMAC scores in older adults with knee osteoarthritis.

Group comparisons were conducted to examine the graded association between the frequency of aerobic and knee strengthening exercise and clinical outcomes related to pain and physical function. Participants engaging in aerobic exercise 3–6 times per week or daily reported significantly lower pain severity compared with non-exercisers (median 2.0 and 2.3 vs. 3.3, *p* = 0.010), lower pain interference (median 1.3 and 1.1 vs. 3.1, *p* = 0.002), and lower total WOMAC scores (median 34.0 and 39.0 vs. 66.0, *p* = 0.001). In detail, higher frequencies of aerobic exercise were also associated with significantly lower WOMAC pain and functional domain scores, whereas no significant difference was observed in the stiffness domain (*p* = 0.758) (Table 2 and Figure 2). In contrast, no significant differences were observed in WOMAC domain scores, pain severity, or pain interference across categories of knee strengthening exercise frequency (Table 2).

### 3.3. Factors Associated with WOMAC Scores

Furthermore, our study results showed that age, BMI, aerobic exercise, and underlying conditions, specifically dyslipidemia and cancer, were significantly associated with total modified WOMAC scores in patients with knee osteoarthritis (*p* < 0.05). The adjusted regression coefficients indicated that being underweight was associated with a decrease in the total modified WOMAC score, whereas being overweight or obese was associated with an increase in the WOMAC scores. Notably, dyslipidemia and cancer were inversely associated with total WOMAC scores in the adjusted model, correlating with lower modified WOMAC scores. In addition, aerobic exercise was significantly associated with lower WOMAC scores (Table 3). The characteristics of older adults with knee osteoarthritis, categorized according to WOMAC score, are presented in Appendix A.

Multivariable regression analysis identified aerobic exercise participation as an independent predictor of lower total WOMAC scores (β = −31.4, 95% CI: −44.6 to −18.3, *p* < 0.001). Overweight combined with obesity was associated with higher WOMAC scores (β = 10.0, 95% CI: 2.8 to 27.9, *p* = 0.017). Conversely, dyslipidemia (β = −15.1, 95% CI: −26.2 to −4.0, *p* = 0.008) and cancer (β = −21.4, 95% CI: −42.6 to −0.3, *p* = 0.047) were associated with lower WOMAC scores.

## 4. Discussion

In this study, we examined the association between the frequency of aerobic and knee-strengthening exercises and the severity of knee osteoarthritis in older adults as measured by composite pain severity, pain interference, and WOMAC scores. The results demonstrated that individuals who engaged in aerobic exercise more frequently reported significantly lower scores on all outcome measures. These findings suggest that regular aerobic exercise may have beneficial effects on pain and functional limitations in older adults with knee osteoarthritis.

The group comparisons of exercise frequency demonstrated significant differences in pain severity, pain interference, and WOMAC scores across varying levels of aerobic exercise, with higher exercise frequency tending to result in lower pain and functional limitation. These results are consistent with those of previous studies highlighting the role of moderate-intensity aerobic activity in improving joint mobility, reducing pain, and enhancing quality of life in individuals with osteoarthritis [4,7]. Regular aerobic exercise has been shown to reduce pain severity and interference in older adults with knee osteoarthritis through several physiological and psychological mechanisms. First, aerobic exercise improves systemic circulation, which facilitates the delivery of oxygen and nutrients to joint tissues, potentially reducing inflammation and promoting cartilage health. Additionally, aerobic activity can stimulate the release of endorphins and other neurochemical mediators that modulate pain perception, contributing to the reduction in chronic pain symptoms [6,16]. Second, exercise-induced neuromodulation enhances central pain inhibitory pathways, thereby decreasing pain interference with daily functions. Furthermore, improved cardiovascular fitness and muscle endurance around the knee joint can lead to better joint stability, reduced mechanical stress on the articular surfaces, and enhanced functional performance. Third, psychosocial benefits, such as improved mood, reduced anxiety, and greater self-efficacy, may further contribute to lower perceived pain and interference with daily activities [17]. Consistent with these mechanisms, our study results showed that pain severity, pain interference, and the pain domain of WOMAC scores were all negatively associated with exercise frequency.

In contrast to aerobic exercise, this study found no significant association between the frequency of knee-strengthening exercise and knee pain outcomes, which may not align with previous studies that demonstrated the effectiveness of strengthening interventions for knee osteoarthritis [9,18]. However, the findings of the present study should be interpreted under several methodological considerations. First, the cross-sectional design and single-time-point data collection limited the ability to capture important characteristics of strengthening exercise, such as exercise type, load, progression, and adherence. The absence of these parameters may have restricted the ability to appropriately classify strengthening exercise exposure and to detect meaningful associations with pain outcomes. In addition, participants in this study exhibited substantial heterogeneity in terms of exercise instruction and guidance. As a tertiary hospital setting involves multiple healthcare professionals providing exercise advice, variations in instructional quality, clarity, and emphasis are likely. Such variability may influence patients’ understanding and execution of strengthening exercises. This interpretation is supported by previous evidence indicating that the effectiveness of exercise interventions is strongly influenced by the quality of instruction and supervision, which directly affects patients’ ability to perform exercises correctly and consistently. Latham et al. suggested that the benefits of resistance training in osteoarthritis populations may be more apparent in long-term interventions, especially when delivered through structured and supervised program [19]. Therefore, from the authors’ perspective, the lack of a detected association in this study is more likely attributable to heterogeneity in exercise delivery and measurement limitations rather than to the ineffectiveness of strengthening exercise itself [4,20,21,22]. In addition, although this study intentionally restricted the strengthening exercise question to activities performed “as recommended by a healthcare professional” in order to ensure that the reported exercises were aligned with established recommendations, this approach may nevertheless have introduced selection or misclassification bias. Individuals engaging in self-initiated strengthening, informal community programmes, or prior instructions not explicitly recognised as professional recommendations may have been classified as non-participants. Consequently, the strengthening group may disproportionately represent participants with greater healthcare access or higher clinical severity, rather than strengthening behavior itself. Future longitudinal studies incorporating standardized exercise prescriptions and monitoring strategies are encouraged to better demonstrate the relationship between resistance frequency and disease progression in knee osteoarthritis.

In the physical function domain, the present study showed that the functional domain of WOMAC was significantly lower in participants who engaged in aerobic exercise at a higher frequency. This finding aligns with those of previous research indicating that regular aerobic exercise positively contributes to joint function and physical capability in older adults with knee osteoarthritis [23,24]. The mechanism underlying this improvement may be related to increased muscle strength and endurance surrounding the knee joint, which enhances joint stability, proprioception, and neuromuscular control as well as reduces mechanical loading on articular surfaces, during functional task movement [25,26]. This observational study revealed that running and walking are the predominant aerobic exercise patterns among older adults with knee osteoarthritis in this cohort. The results indicated that an exercise frequency of 3–6 times per week was most strongly associated with a reduction in pain severity, pain interference, and functional limitations. This relationship aligns with the existing literature proposing that moderate-to high-frequency aerobic activities such as walking and running offer substantial benefits for symptom management in knee osteoarthritis through mechanisms including improved joint lubrication, enhanced muscular support, and reduced inflammation [24,25]. Furthermore, the beneficial effects observed with daily aerobic exercise may be explained by the consistent activation of neuromodulator pathways that promote pain relief through endogenous analgesic release and improved systemic circulation [26]. Notably, exercising 3–6 times per week was associated with lower pain and functional limitation than daily exercise. Exercise-induced hypoalgesia depends on both stimulus intensity and adequate recovery. Although aerobic exercise activates endogenous analgesic mechanisms, including central pain inhibitory pathways and endorphin release, insufficient recovery with daily repetition may attenuate these effects or transiently exacerbate symptoms in individuals with chronic musculoskeletal pain. This may partly explain the slightly higher pain severity and interference observed in the daily exercise group compared with the 3–6 times per week group, despite better overall benefits relative to non-exercisers. Therefore, this study supports the incorporation of regular aerobic exercises, performed for 3–6 times weekly, into therapeutic regimens aimed at optimizing pain relief and functional improvement in older adults with knee osteoarthritis. Moreover, our results showed that BMI was significantly positively associated with the severity of knee osteoarthritis, as measured using the modified WOMAC. This may be attributed to the fact that 69.29% of participants in this study were overweight and obese across all age groups, which may induce pathological changes in the whole knee joint structure, including abnormal loading on the joint, joint malalignment, and muscle weakness. The combination of these three mechanical factors affects joint structure and stimulates the onset and progression of overweight-related osteoarthritis [27]. Further, many previous studies have shown an association between higher BMI and increased knee osteoarthritis severity [28,29].

An inverse association between dyslipidemia, cancer, and total WOMAC scores was observed in the adjusted analyses. This may be explained by previous evidence that reported statins and HMG-CoA reductase inhibitors used in the management of dyslipidemia may be associated with reduced osteoarthritic pain and modulation of inflammatory pathways in joint tissues [30]. In addition, pain management and altered activity patterns in individuals with cancer have been reported to influence musculoskeletal pain perception [31]. These findings from prior studies provide a possible contextual framework for the inverse associations observed in the present analysis. However, given the cross-sectional design of this study and the absence of detailed information on medication use, disease severity, and treatment characteristics, which are reported as important factors in treating musculoskeletal pain in older adults [32], as well as the small sizes of participants in some subgroups, these interpretations remain speculative.

Several methodological considerations should be acknowledged. First, the cross-sectional design precludes causal inference regarding the effects of exercise frequency on symptom changes over time. Second, exercise behaviors were self-reported, which may introduce recall and reporting bias. Third, although key covariates were adjusted for, residual confounding cannot be fully excluded, as information on disease duration, radiographic severity, analgesic use, and other contextual factors was not comprehensively captured. In addition, detailed exercise prescription parameters, such as specific patterns, sets, or repetitions, were not assessed, potentially contributing to heterogeneity in exercise exposure. Participants also received exercise advice from different healthcare professionals across clinical settings, which may have varied in content and emphasis and influenced adherence and execution. Finally, as participants were recruited from a tertiary care hospital, the generalizability of the findings to other populations and healthcare settings may be limited. Despite these considerations, the observed associations highlight the potential relevance of regular aerobic exercise in the management of knee osteoarthritis and provide clinically meaningful insights to inform future longitudinal and interventional studies.

## 5. Conclusions

This study identified a graded association between the frequency of aerobic exercise and pain severity, pain interference, and knee-related functional outcomes among older adults with knee osteoarthritis. Aerobic activities such as walking and running performed at higher frequencies, particularly 3–6 times per week or every day, were associated with lower pain severity, reduced pain interference in daily activities, and lower knee-related physical function. These findings support the clinical importance of emphasizing exercise frequency when prescribing aerobic exercise for pain management in knee osteoarthritis.

## Figures and Tables

**Table 1:** Baseline characteristics of the study participants according to knee pain severity category (NPRS) (N = 140).

Variables	Knee Pain Severity (NPRS)		
	Mild n = 58	Moderate n = 72	Severe n = 10
Sex, N (%)			
Male (n = 48)	21 (43.8)	24 (50.0)	3 (6.3)
Female (n = 92)	37 (40.2)	48 (52.2)	7 (7.6)
Age, N (%)			
60–64 (n = 25)	12 (48.0)	12 (48.0)	1 (4.0)
65–69 (n = 43)	19 (44.2)	23 (53.5)	1 (2.3)
70–74 (n = 30)	12 (40.0)	15 (50.0)	3 (10.0)
75–80 (n = 27)	12 (44.4)	12 (44.4)	3 (11.1)
>80 (n = 15)	3 (20.0)	10 (66.7)	2 (13.3)
BMI (kg/m^2^), N (%)			
Underweight (<18.5) (n = 4)	2 (50.0)	2 (50.0)	0 (0.0)
Normal (18.5–22.9) (n = 39)	17 (43.6)	20 (51.3)	2 (5.1)
Overweight (23–27.5) (n = 89)	23 (25.8)	42 (47.2)	24 (27.0)
Obesity (≥27.5) (n = 8)	1 (12.5)	4 (50.0)	3 (37.5)
Marital status, N (%)			
Single (n = 9)	4 (44.4)	4 (44.4)	1 (11.1)
Married (n = 102)	42 (41.2)	53 (52.0)	7 (6.9)
Divorced/widow (n = 29)	12 (41.4)	15 (51.7)	2 (6.9)
Education levels, N (%)			
Primary school (n = 7)	2 (28.6)	5 (71.4)	0 (0.0)
High school (n = 15)	5 (33.3)	9 (60.0)	1 (6.7)
Occupational/Technical school (n = 22)	6 (27.3)	13 (59.1)	3 (13.6)
Bachelor’s degree (n = 43)	25 (58.1)	16 (37.2)	2 (4.7)
Postgraduation degree (n = 10)	7 (70.0)	2 (20.0)	1 (10.0)
Other (n = 43)	13 (30.2)	27 (62.8)	3 (7.0)
Underlying disease, N (%)			
Hypertension (n = 71)	28 (39.4)	34 (47.9)	9 (12.7)
Dyslipidemia (n = 72)	38 (52.8)	28 (38.9)	6 (8.3)
Diabetes (n = 36)	12 (33.3)	18 (50.0)	6 (16.7)
Cancers (n = 10)	3 (30.0)	7 (70.0)	0 (0.0)
CVD (n = 9)	7 (77.8)	2 (22.2)	0 (0.0)
CKD (n = 7)	1 (14.3)	5 (71.4)	1 (14.3)
Smoking habit, N (%)			
No (n = 126)	51 (40.5)	66 (52.4)	9 (7.1)
Yes (n = 14)	7 (50.0)	6 (42.9)	1 (7.1)
Drinking habit, N (%)			
No (n = 126)	53 (42.1)	63 (50.0)	10 (7.9)
Yes (n = 14)	5 (35.7)	9 (64.3)	0 (0.0)

BMI, body mass index; CKD, chronic kidney disease; CVD, cardiovascular disease.

**Table 2:** Pain severity, pain interference, and WOMAC scores by frequency of aerobic and knee strengthening exercise among patients with knee osteoarthritis.

Variables	Frequency of Exercise (Times per Week)				*p*-Value
	None	1–2	3–6	Every Day	
**Aerobic exercise**					
Brief Pain Inventory (median (IQR))					
Pain severity (0–10 scores)	3.3 (2.5, 4.5)	3.3 (2.1, 4.5)	2.0 (0.9, 3.8)	2.3 (1.4, 3.8)	0.010 *
Pain interference (0–10 scores)	3.1 (1.6, 4.4)	2.1 (0.7, 3.8)	1.3 (0.3, 2.4)	1.1 (0.4, 3.4)	0.002 *
WOMAC scores (median (IQR))					
Pain domain (0–50 scores)	12.0 (9.0, 16.0)	9.0 (5.0, 16.0)	8.0 (3.5, 14.5)	8.0 (4.0, 13.0)	0.010 *
Stiffness domain (0–20 scores)	0.0 (0.0, 3.0)	0.0 (0.0, 5.0)	0.0 (0.0, 4.5)	0.0 (0.0, 3.0)	0.758
Functional domain (0–170 scores) Total WOMAC scores (0–240 scores)	43.0 (26.0, 68.0) 66.0 (44.0, 88.0)	25.0 (11.5, 50.0) 38.0 (25.0, 70.0)	20.0 (6.0, 41.5) 34.0 (18.0, 64.0)	21.0 (11.0, 40.5) 39.0 (22.0, 57.5)	0.011 * 0.001 *
**Knee strengthening exercise**					
Brief Pain Inventory (median (IQR))					
Pain severity (0–10 scores)	2.5 (1.8, 3.0)	2.8 (1.0, 4.0)	3.1 (1.9, 3.8)	3.3 (2.3, 4.5)	0.628
Pain interference (0–10 scores)	1.6 (0.7, 3.6)	2.6 (0.7, 4.4)	1.7 (0.6, 3.2)	2.1 (0.8, 3.4)	0.548
WOMAC scores (median (IQR))					
Pain domain (0–50 scores)	9.0 (5.0, 13.0)	11.0 (5.0, 22.0)	9.0 (6.5, 14.5)	11.0 (3.3, 16.8)	0.474
Stiffness domain (0–20 scores)	0.0 (0.0, 3.5)	0.0 (0.0, 4.0)	0.0 (0.0, 4.3)	0.0 (0.0, 1.75)	0.776
Functional domain (0–170 scores) Total WOMAC scores (0–240 scores)	26.0 (13.0, 50.0) 42.0 (23.0, 68.0)	32.0 (20.0, 43.0) 55.0 (28.0, 73.0)	31.5 (17.8, 46.5) 48.5 (28.3, 71.5)	33.5 (0.0, 51.0) 48.0 (21.5, 77.3)	0.713 0.664

* Significant difference between groups (*p* < 0.05). WOMAC, Western Ontario and McMaster Universities Osteoarthritis Index.

**Table 3:** Multivariable regression analysis of factors associated with knee osteoarthritis severity based on total WOMAC scores (N = 140).

Variables	Adjusted Coefficient	95% CI	*p*-Value *t*-Test
Age (reference: 60–64)			
65–69 (n = 43)	−13.3	(−29.4, 2.8)	0.105
70–74 (n = 30)	−7.8	(−25.3, 9.8)	0.381
75–80 (n = 27)	−0.7	(−19.1, 17.8)	0.941
>80 (n = 15)	16.3	(−4.9, 37.4)	0.13
BMI (reference: normal)			
Underweight (n = 4)	−11.5	(−53.7, 14.1)	0.25
Overweight and obesity (n = 97)	10	(2.8, 27.9)	0.017 *
Aerobic exercise participation	−31.4	(−44.6, −18.3)	<0.001 *
(reference: no exercise)			
Underlying disease			
Dyslipidemia (n = 72)	−15.1	(−26.2, −4.1)	0.008 *
Cancer (n = 10)	−21.4	(−42.6, −0.3)	0.047 *

Only comorbidities retained in the final multivariable model are presented in Table 3. * Significant difference between groups (*p* < 0.05). Adjusted for age, BMI, underlying disease, aerobic exercise, and knee-strengthening exercise. BMI, body mass index; CI, confidence interval; WOMAC, Western Ontario and McMaster Universities Osteoarthritis Index.

## Data Availability

The data that support the findings of this study are available from the corresponding author upon reasonable request. The data are not publicly available due to privacy and ethical restrictions.

## Abbreviations

The following abbreviations are used in this manuscript:

ADL	Activities of Daily Living
BMI	Body Mass Index
BPI	Brief Pain Inventory
BPI-T	Thai Version of the Brief Pain Inventory
CI	Confidence Interval
CKD	Chronic Kidney Disease
CVD	Cardiovascular Disease
IQR	Interquartile Range
OA	Osteoarthritis
REC	Research Ethics Committee
SD	Standard Deviation
WOMAC	Western Ontario and McMaster Universities Osteoarthritis Index

## Appendix A

**Table A1:** Modified WOMAC scores categorized by participants’ characteristics in elderly with Knee Osteoarthritis (n = 140).

Factors	Modified WOMAC Scores							
	Sum Score of Pain Domain	*p*-Value	Sum Score of Stiffness Domain	*p*-Value	Sum Score of Functional Domains	*p*-Value	Sum of Total Modified WOMAC	*p*-Value
Sarcopenia risk, median (IQR) -No (n = 102) -Yes (n = 38)	10.0 (5.00, 15.00) 9.0 (5.00, 14.80)	0.771 ^w^	0.0 (0.00, 4.00) 0.0 (0.00, 3.00)	0.989 ^w^	32.0 (13.50, 51.00) 32.0 (14.80, 57.50)	0.996 ^w^	52 (23.20, 73.00) 45.5 (26.20, 79.80)	0.918 ^w^
Gender, median (IQR) -Female (n = 92) -Male (n = 48)	9.5 (5.00, 14.50) 10.0 (5.00, 15.25)	0.855 ^w^	0.0 (0.00, 4.00) 0.0 (0.00, 3.00)	0.476 ^w^	33.0 (17.75, 1.00) 28.0 (11.75, 59.25)	0.628 ^w^	50.5 (25.75, 72.25) 48 (20.00, 80.75)	0.562 ^w^
Age, median (IQR) -60–64 (n = 25) -65–69 (n = 43) -70–74 (n = 30) -75–80 (n = 27) ->80 (n = 15)	9.0 (5.00, 16.00) 9.0 (5.00, 15.00) 9.5 (4.25, 14.75) 9.0 (4.50, 15.00) 12.0 (9.00, 15.50)	0.720 ^k^	2.0 (0.00, 5.00) 0.0 (0.00, 4.00) 0.0 (0.00, 2.25) 0.0 (0.00, 0.00) 0.0 (0.00, 5.50)	0.117 ^k^	28.0 (11.00, 64.00) 32.0 (13.00, 44.00) 28.0 (13.00, 42.50) 35.0 (16.00, 57.00) 47.0 (28.00, 74.00)	0.341 ^k^	42.0 (24.00, 83.00) 47.0 (25.00, 64.50) 42.0 (21.25, 72.50) 53.0 (26.00, 79.50) 68.0 (46.50, 88.50)	0.310 ^k^
BMI (kg/m^2^), median (IQR) -Underweight (<18.5) (n = 4) -Normal (18.5–22.9) (n = 39) -Obese (>22.9) (n = 97)	8.0 (1.50, 14.50) 9.0 (4.00, 13.50) 10 (6.00, 16.00)	0.419 ^k^	0.0 (0.00, 0.00) 0.0 (0.00, 2.00) 0.0 (0.00, 4.00)	0.235 ^k^	9.0 (4.50, 24.20) 25.0 (7.50, 42.50) 36.0 (18.00, 56.00)	* 0.027 ^k^	22.0 (8.20, 45.20) 37.0 (18.00, 69.00) 55.0 (28.00, 80.00)	* 0.049 ^k^
Marital status, median (IQR) -Single (n = 9) -Married (n = 102) -Divorced/widow (n = 29)	9.0 (6.00, 10.00) 10.0 (5.00, 15.00) 10.0 (5.00, 16.00)	0.545 ^k^	0.0 (0.00, 4.00) 0.0 (0.00, 4.75) 0.0 (0.00, 3.00)	0.936 ^k^	32.0 (7.00, 47.00) 32.0 (13.25, 55.50) 33.0 (18.00, 48.00)	0.720 ^k^	46 (18.00, 64.00) 45 (24.25, 79.00) 52 (30.00, 73.00)	0.644 ^k^
Education levels, median (IQR) -Junior high school (n = 7) -Senior high school (n = 15) -Vocational/Technical school (n = 22) -Bachelor’s degree (n = 43) -Master’s/Doctoral degree (n = 10) -Others (n = 43)	16.0 (7.00, 17.50) 10.0 (7.00, 14.50) 16.0 (5.00, 21.50) 7.0 (4.00, 12.00) 8.5 (4.00, 9.75) 11.0 (7.00, 15.00)	* 0.033 ^k^	0.0 (0.00, 2.00) 0.0 (0.00, 0.00) 0.0 (0.00, 6.50) 0.0 (0.00, 3.00) 0.0 (0.00, 3.25) 0.0 (0.00, 4.50)	0.222 ^k^	38.0 (32.50, 42.50) 30.0 (16.00, 45.00) 34.5 (13.00, 60.25) 21.0 (9.50, 42.00) 28.0 (11.25, 43.25) 41.0 (20.50, 66.50)	* 0.049 ^k^	56.0 (53.50, 59.50) 48.0 (21.50, 67.00) 54.0 (28.25, 85.50) 34.0 (21.00, 62.50) 36.0 (17.75, 61.75) 67.0 (40.50, 86.50)	* 0.030 ^k^
Underlying disease, median (IQR) -Hypertension (n = 71) -Diabetes (n = 36) -Dyslipidemia (n = 72) -Cancers (n = 10) -CVD (n = 9) -CKD (n = 7)	10.0 (5.00, 14.50) 10.5 (4.75, 16.00) 8.5 (4.00, 13.25) 5.0 (3.25, 10.75) 5.0 (2.00, 14.00) 16.0 (12.50, 17.00)	0.770 ^w^ 0.513 ^w^ * 0.039 ^w^ 0.072 ^w^ 0.115 ^w^ 0.109 ^w^	0.0 (0.00, 3.00) 0.0 (0.00, 3.00) 0.0 (0.00, 3.00) 2.0 (0.00, 4.75) 0.0 (0.00, 0.00) 0.0 (0.00, 4.50)	0.850 ^w^ 0.920 ^w^ 0.432 ^w^ 0.279 ^w^ 0.364 ^w^ 0.734 ^w^	35.0 (16.00, 60.50) 38.5 (20.00, 62.25) 20.5 (10.00, 43.50) 15.5 (8.50, 39.25) 25.0 (12.00, 35.00) 38.0 (18.00, 78.00)	0.216 ^w^ 0.06 ^w^ * 0.002 ^w^ 0.137 ^w^ 0.427 ^w^ 0.602 ^w^	55.0 (26.50, 83.50) 59.0 (27.75, 86.25) 37.0 (20.00, 63.75) 30.5 (18.75, 49.25) 28.0 (20.00, 55.00) 71.0 (41.50, 96.00)	0.304 ^w^ 0.094 ^w^ * 0.003 ^w^ 0.111 ^w^ 0.236 ^w^ 0.334 ^w^
Smoking habit, median (IQR) -No (n = 126) -Yes (n = 14)	10.0 (5.00, 15.00) 10.0 (4.25, 15.25)	0.723 ^w^	0.0 (0.00, 4.00) 0.0 (0.00, 4.00)	0.984 ^w^	32.0 (13.25, 51.00) 42.0 (17.50, 61.50)	0.380 ^w^	50.5 (24.25, 71.75) 60.0 (22.00, 85.50)	0.657 ^w^
Drinking habit, median (IQR) -No (n = 126) -Yes (n = 14)	9.5 (5.00, 15.00) 11.0 (4.25, 12.75)	0.920 ^w^	0.0 (0.00, 4.00) 0.0 (0.00, 4.00)	0.874 ^w^	32.5 (15.00, 51.00) 25.5 (8.75, 63.50)	0.950 ^w^	52.0 (24.25, 72.75) 42.5 (20.25, 83.75)	0.986 ^w^

* Significant difference between groups (*p* < 0.05); ^w^ Wilcoxon rank-sum test, ^k^ Kruskal–Wallis test.

## References

[1] Bannuru R.R., Osani M.C., Vaysbrot E.E., Arden N.K., Bennell K., Bierma-Zeinstra S.M.A., Kraus V.B., Lohmander L.S., Abbott J.H., Bhandari M. (2019). OARSI guidelines for the non-surgical management of knee, hip, and polyarticular osteoarthritis. Osteoarthr. Cartil..

[2] Hunter D.J., Bierma-Zeinstra S.M.A. (2019). Osteoarthritis. Lancet.

[3] Vos T., Lim S.S., Abbafati C., Abbas K.M., Abbasi M., Abbasifard M., Abbasi-Kangevari M., Abbastabar H., Abd-Allah F., Abdelalim A. (2020). Global burden of 369 diseases and injuries in 204 countries and territories, 1990–2019: A systematic analysis for the Global Burden of Disease Study 2019. Lancet.

[4] Fransen M., McConnell S., Harmer A.R., van der Esch M., Simic M., Bennell K.L. (2015). Exercise for osteoarthritis of the knee: A Cochrane systematic review. Br. J. Sports Med..

[5] Messier S.P. (2012). Effects of exercise interventions in older adults with knee osteoarthritis. HSS J..

[6] Geneen L.J., Moore R.A., Clarke C., Martin D., Colvin L.A., Smith B.H. (2017). Physical activity and exercise for chronic pain in adults: An overview of Cochrane Reviews. Cochrane Database Syst. Rev..

[7] Bennell K.L., Hunt M.A., Wrigley T.V., Lim B.-W., Hinman R.S. (2008). Role of muscle in the genesis and management of knee osteoarthritis. Rheum. Dis. Clin. N. Am..

[8] Lange A.K., Vanwanseele B., Fiatarone Singh M.A. (2008). Strength training for treatment of osteoarthritis of the knee: A systematic review. Arthritis Care Res..

[9] Juhl C., Christensen R., Roos E.M., Zhang W., Lund H. (2014). Impact of exercise type and dose on pain and disability in knee osteoarthritis: A systematic review and meta-regression analysis of randomized controlled trials. Arthritis Rheumatol..

[10] Cohen J. (2013). Statistical Power Analysis for the Behavioral Sciences.

[11] Borg G.A. (1982). Psychophysical bases of perceived exertion. Med. Sci. Sports Exerc..

[12] Kuptniratsaikul V., Rattanachaiyanont M. (2007). Validation of a modified Thai version of the Western Ontario and McMaster Universities Osteoarthritis Index (WOMAC) for knee osteoarthritis. Clin. Rheumatol..

[13] Chaudakshetrin P. (2009). Validation of the Thai version of the Brief Pain Inventory (BPI-T) in cancer patients. J. Med. Assoc. Thai..

[14] Department of Mental Health, Ministry of Public Health (2016). 2Q, 9Q, 8Q Screening Questionnaires.

[15] Jih J., Mukherjea A., Vittinghoff E., Nguyen T.T., Tsoh J.Y., Fukuoka Y., Bender M.S., Tseng W., Kanaya A.M. (2014). Using appropriate body mass index cut points for overweight and obesity among Asian Americans. Prev. Med..

[16] Wu P., Chen X., Wang S., Chen X., Liu J. (2025). Effects of exercise on depression and anxiety in patients with chronic pain: A systematic review and meta-analysis of randomized controlled trials. J. Affect. Disord..

[17] Edwards R.R., Dworkin R.H., Sullivan M.D., Turk D.C., Wasan A.D. (2016). The role of psychosocial processes in the development and maintenance of chronic pain. J. Pain.

[18] Chuchuen P., Yuenyongviwat V., Hongnaparak T., Iamthanaporn K., Geater A., Chotipanvithayakul R., Mankaket J., Limsakul C. (2024). The effect of a single session of preoperative exercise training on functional improvement after total knee arthroplasty. J. Health Sci. Med. Res..

[19] Latham N., Liu C.J. (2010). Strength training in older adults: The benefits for osteoarthritis. Clin. Geriatr. Med..

[20] Brosseau L., Wells G.A., Pugh A.G., Smith C.A., Rahman P., Álvarez Gallardo I.C., Toupin-April K., Loew L., De Angelis G., Cavallo S. (2016). Ottawa Panel evidence-based clinical practice guidelines for therapeutic exercise in hip osteoarthritis. Clin. Rehabil..

[21] Goh S.-L., Persson M.S.M., Stocks J., Hou Y., Welton N.J., Lin J., Hall M.C., Doherty M., Zhang W. (2019). Relative efficacy of different exercises for pain and function in knee and hip osteoarthritis: A systematic review and network meta-analysis. Sports Med..

[22] Morris L., Moule P., Pearson J., Foster D., Walsh N. (2021). Patient views of the advanced practitioner role in primary care. Musculoskelet. Care.

[23] Hurley M., Dickson K., Hallett R., Grant R., Hauari H., Walsh N., Stansfield C., Oliver S. (2018). Exercise interventions and patient beliefs for hip and knee osteoarthritis: A mixed-methods review. Cochrane Database Syst. Rev..

[24] Raposo F., Ramos M., Cruz A.L. (2021). Effects of exercise on knee osteoarthritis: A systematic review. Musculoskelet. Care.

[25] Mo L., Jiang B., Mei T., Zhou D. (2023). Exercise therapy for knee osteoarthritis: A systematic review and network meta-analysis. Orthop. J. Sports Med..

[26] Vaegter H.B., Jones M.D. (2020). Exercise-induced hypoalgesia after acute and regular exercise. Pain Rep..

[27] Chen L., Zheng J.J.Y., Li G., Yuan J., Ebert J.R., Li H., Papadimitriou J., Wang Q., Wood D., Jones C.W. (2020). Pathogenesis and clinical management of obesity-related knee osteoarthritis. J. Orthop. Transl..

[28] Elbaz A., Debbi E.M., Segal G., Haim A., Halperin N., Agar G., Mor A., Debi R. (2011). Sex and body mass index correlate with WOMAC and quality of life in knee osteoarthritis. Arch. Phys. Med. Rehabil..

[29] Rawdha T., Aicha B.T., Leila R., Olfa S., Selma B., Ines M., Leila A. (2023). Correlation between abdominal obesity and pain in knee osteoarthritis. Curr. Rheumatol. Rev..

[30] Saberianpour S., Abolbashari S., Modaghegh M.H., Karimian M.S., Eid A.H., Sathyapalan T., Sahebkar A. (2022). Therapeutic effects of statins on osteoarthritis. J. Cell. Biochem..

[31] Maddocks M. (2020). Physical activity and exercise training in cancer patients. Clin. Nutr. ESPEN.

[32] Srikrajang S., Komolsuradej N., Chaovalit S., Chuaychoosakoon C. (2024). Effects of the WHO analgesic ladder on pain outcomes in patients with chronic musculoskeletal pain. Prim. Health Care Res. Dev..

